# Supplementary material for: A Likelihood Approach for Real-Time Calibration of Stochastic Compartmental Epidemic Models
Source: PLoS Comput Biol. 2017 Jan 17;13(1):e1005257. doi: 10.1371/journal.pcbi.1005257 (PMC5240920; doi:10.1371/journal.pcbi.1005257)
Supplement: S1 File — (TAR.GZ) [file pcbi.1005257.s014.tar.gz › HSPH_Online-SI-Revision/MSS10/n1-severe/n1-severe_NEW2_table-4.pdf]

1	newly 3 weeks spec	33.	24.11	{0., 58.}	69.1818	0.761633	newly 3 weeks cumul	81.	98.08	{19., 201.}	69.8519	0.328497	newly 5 weeks spec	34.	10.68	{0., 33.}	76.1765	1.46993	newly 5 weeks cumul	158.	125.25	{19., 258.}	55.3987	0.398885	newly 58 weeks cumul	531.	142.57	{19., 298.}	73.1507	0.777619
2	newly 3 weeks spec	55.	30.97	{0., 67.}	57.5455	0.674847	newly 3 weeks cumul	130.	89.26	{19., 171.}	50.3846	0.376978	newly 5 weeks spec	53.	25.85	{0., 58.}	60.0943	0.924128	newly 5 weeks cumul	247.	146.66	{22., 303.}	51.7328	0.443554	newly 60 weeks cumul	592.	268.82	{22., 533.}	55.3446	0.571297
3	newly 3 weeks spec	32.	23.68	{0., 55.}	63.375	0.777414	newly 3 weeks cumul	74.	85.42	{17., 167.}	63.2162	0.324351	newly 5 weeks spec	43.	16.33	{0., 41.}	67.9302	1.2255	newly 5 weeks cumul	164.	120.85	{17., 228.}	47.1402	0.379563	newly 59 weeks cumul	553.	181.75	{17., 388.}	67.1338	0.706372
4	newly 3 weeks spec	58.	54.77	{30., 81.}	28.6379	0.141345	newly 3 weeks cumul	144.	122.24	{74., 175.}	26.1111	0.138487	newly 5 weeks spec	59.	76.27	{51., 98.}	37.0678	0.139425	newly 5 weeks cumul	286.	266.65	{174., 352.}	21.1503	0.10431	newly 62 weeks cumul	620.	777.98	{686., 865.}	25.6839	0.0977121
5	newly 3 weeks spec	56.	83.7	{60., 105.}	51.8571	0.174251	newly 3 weeks cumul	140.	203.73	{155., 253.}	46.5929	0.160136	newly 5 weeks spec	40.	85.88	{71., 102.}	114.7	0.326594	newly 5 weeks cumul	241.	378.69	{301., 449.}	57.1328	0.190723	newly 61 weeks cumul	534.	769.74	{692., 848.}	44.1461	0.15733
6	newly 3 weeks spec	31.	17.46	{7., 29.}	49.4194	0.34361	newly 3 weeks cumul	71.	44.97	{22., 75.}	42.5775	0.270481	newly 5 weeks spec	40.	22.64	{10., 38.}	46.55	0.320463	newly 5 weeks cumul	159.	88.09	{40., 140.}	47.7421	0.318434	newly 58 weeks cumul	620.	527.63	{331., 735.}	22.1403	0.120883
7	newly 3 weeks spec	37.	56.6	{37., 76.}	56.7568	0.186345	newly 3 weeks cumul	101.	154.1	{103., 196.}	54.5941	0.181522	newly 5 weeks spec	30.	57.01	{39., 75.}	92.4333	0.275417	newly 5 weeks cumul	170.	268.19	{189., 332.}	59.2529	0.19443	newly 58 weeks cumul	392.	578.16	{454., 657.}	48.9286	0.169793
8	newly 3 weeks spec	83.	62.14	{35., 87.}	29.5422	0.168399	newly 3 weeks cumul	181.	148.61	{97., 203.}	24.7238	0.131903	newly 5 weeks spec	97.	72.79	{46., 94.}	25.9897	0.147119	newly 5 weeks cumul	384.	290.96	{182., 381.}	25.9583	0.14348	newly 62 weeks cumul	588.	689.37	{592., 802.}	18.7364	0.0729093
9	newly 3 weeks spec	38.	33.51	{22., 49.}	29.6579	0.148455	newly 3 weeks cumul	79.	86.08	{59., 121.}	24.557	0.0984483	newly 5 weeks spec	31.	39.79	{25., 55.}	40.4839	0.170975	newly 5 weeks cumul	150.	163.46	{108., 215.}	25.12	0.103625	newly 58 weeks cumul	454.	585.38	{503., 697.}	34.1982	0.138224
10	newly 3 weeks spec	67.	74.07	{57., 90.}	17.1791	0.0686221	newly 3 weeks cumul	156.	195.6	{163., 232.}	26.1538	0.0976896	newly 5 weeks spec	47.	71.26	{57., 83.}	51.617	0.176122	newly 5 weeks cumul	273.	341.68	{287., 385.}	25.3993	0.0959916	newly 58 weeks cumul	540.	702.73	{646., 742.}	30.1352	0.113804
11	newly 3 weeks spec	25.	31.59	{17., 45.}	40.12	0.148499	newly 3 weeks cumul	76.	86.21	{59., 119.}	25.4605	0.102492	newly 5 weeks spec	31.	35.61	{19., 53.}	36.0323	0.151449	newly 5 weeks cumul	146.	155.94	{96., 211.}	23.9178	0.10292	newly 55 weeks cumul	505.	429.29	{264., 526.}	16.6277	0.0957654
12	newly 3 weeks spec	95.	109.39	{85., 136.}	19.7789	0.0765217	newly 3 weeks cumul	232.	260.19	{206., 320.}	17.2026	0.0679813	newly 5 weeks spec	59.	104.35	{89., 117.}	76.8644	0.245016	newly 5 weeks cumul	391.	479.87	{419., 556.}	23.0563	0.0878674	newly 63 weeks cumul	569.	816.56	{731., 873.}	43.5079	0.155868
13	newly 3 weeks spec	46.	58.3	{44., 72.}	30.3043	0.113061	newly 3 weeks cumul	111.	148.9	{117., 174.}	36.0541	0.130328	newly 5 weeks spec	49.	70.1	{55., 86.}	44.9388	0.156986	newly 5 weeks cumul	221.	284.37	{236., 333.}	30.3484	0.112836	newly 58 weeks cumul	585.	730.77	{655., 800.}	25.2291	0.0965946
14	newly 3 weeks spec	59.	81.77	{45., 116.}	48.661	0.178213	newly 3 weeks cumul	122.	178.99	{101., 253.}	55.2049	0.1904	newly 5 weeks spec	75.	98.04	{74., 119.}	36.16	0.157774	newly 5 weeks cumul	272.	374.22	{247., 486.}	44.2132	0.164729	newly 63 weeks cumul	614.	810.62	{719., 891.}	34.7362	0.140893
15	newly 3 weeks spec	43.	47.76	{32., 62.}	26.6512	0.1202	newly 3 weeks cumul	132.	143.17	{104., 175.}	19.4167	0.0851243	newly 5 weeks spec	36.	41.53	{22., 58.}	31.4722	0.137878	newly 5 weeks cumul	229.	230.51	{167., 286.}	15.6987	0.07601	newly 57 weeks cumul	373.	441.81	{288., 560.}	27.5684	0.110403
16	newly 3 weeks spec	37.	64.67	{30., 105.}	85.5946	0.265577	newly 3 weeks cumul	113.	148.5	{66., 233.}	47.9469	0.178618	newly 5 weeks spec	55.	83.86	{52., 110.}	59.7818	0.206603	newly 5 weeks cumul	226.	311.21	{167., 455.}	49.6416	0.181049	newly 63 weeks cumul	577.	724.67	{523., 860.}	32.896	0.127591
17	newly 3 weeks spec	80.	59.34	{6., 112.}	47.5	0.478168	newly 3 weeks cumul	176.	177.02	{50., 277.}	43.9659	0.272252	newly 5 weeks spec	63.	48.16	{0., 103.}	61.1429	0.74022	newly 5 weeks cumul	335.	279.55	{50., 473.}	42.6119	0.322324	newly 63 weeks cumul	583.	436.39	{50., 830.}	52.4408	0.430912
18	newly 3 weeks spec	27.	53.65	{28., 80.}	101.	0.273519	newly 3 weeks cumul	67.	116.78	{65., 171.}	77.9701	0.228676	newly 5 weeks spec	23.	74.38	{39., 101.}	223.391	0.484114	newly 5 weeks cumul	126.	258.39	{149., 363.}	106.373	0.290511	newly 63 weeks cumul	593.	776.66	{661., 881.}	31.882	0.118449
19	newly 3 weeks spec	46.	31.46	{3., 59.}	47.	0.43395	newly 3 weeks cumul	143.	103.67	{40., 168.}	39.2797	0.250765	newly 5 weeks spec	45.	24.05	{0., 50.}	54.4222	0.748696	newly 5 weeks cumul	247.	155.41	{45., 270.}	42.1822	0.301101	newly 56 weeks cumul	533.	287.73	{45., 537.}	47.3827	0.395442
20	newly 3 weeks spec	63.	64.36	{44., 78.}	15.7778	0.070009	newly 3 weeks cumul	168.	179.86	{132., 206.}	14.4286	0.0615424	newly 5 weeks spec	55.	61.16	{46., 74.}	19.6	0.0811863	newly 5 weeks cumul	291.	305.22	{228., 342.}	12.5979	0.055069	newly 56 weeks cumul	494.	650.51	{546., 703.}	32.1194	0.119471
21	newly 3 weeks spec	38.	62.09	{25., 87.}	79.2895	0.318879	newly 3 weeks cumul	97.	165.94	{89., 240.}	81.4845	0.274738	newly 5 weeks spec	50.	59.62	{13., 95.}	52.52	0.328271	newly 5 weeks cumul	199.	289.61	{156., 398.}	59.4623	0.239903	newly 63 weeks cumul	538.	550.63	{293., 764.}	31.277	0.194946
22	newly 3 weeks spec	77.	46.49	{30., 64.}	39.7013	0.237474	newly 3 weeks cumul	210.	120.87	{89., 151.}	42.6429	0.253776	newly 5 weeks spec	61.	51.99	{33., 69.}	21.918	0.11434	newly 5 weeks cumul	359.	223.22	{162., 275.}	37.8217	0.216364	newly 57 weeks cumul	537.	591.82	{403., 692.}	22.879	0.0981289
23	newly 3 weeks spec	30.	24.8	{0., 52.}	61.2	0.630641	newly 3 weeks cumul	92.	88.29	{10., 166.}	50.7065	0.31146	newly 5 weeks spec	37.	15.26	{0., 40.}	65.5676	1.07593	newly 5 weeks cumul	174.	124.37	{10., 234.}	47.6379	0.384072	newly 59 weeks cumul	461.	177.21	{10., 345.}	64.8351	0.652326
24	newly 3 weeks spec	38.	14.09	{0., 37.}	68.7105	0.974267	newly 3 weeks cumul	121.	58.7	{12., 120.}	56.0992	0.475803	newly 5 weeks spec	32.	6.25	{0., 21.}	80.9688	1.56523	newly 5 weeks cumul	201.	73.23	{12., 156.}	64.0149	0.603972	newly 56 weeks cumul	407.	84.74	{12., 179.}	79.1794	0.865626
25	newly 3 weeks spec	55.	33.07	{4., 60.}	46.6364	0.490389	newly 3 weeks cumul	180.	118.05	{41., 197.}	40.5167	0.295374	newly 5 weeks spec	62.	20.89	{0., 48.}	66.3065	0.87373	newly 5 weeks cumul	314.	165.99	{48., 255.}	47.8312	0.37488	newly 54 weeks cumul	547.	266.05	{49., 603.}	55.0841	0.47433
26	newly 3 weeks spec	41.	86.42	{61., 112.}	113.171	0.326798	newly 3 weeks cumul	123.	216.63	{161., 279.}	78.4634	0.250075	newly 5 weeks spec	42.	86.75	{71., 102.}	108.595	0.337086	newly 5 weeks cumul	210.	393.9	{315., 474.}	89.8381	0.282238	newly 61 weeks cumul	500.	757.76	{704., 824.}	53.488	0.196729
27	newly 3 weeks spec	55.	28.56	{0., 68.}	64.9455	0.860088	newly 3 weeks cumul	170.	105.76	{17., 204.}	50.9294	0.41197	newly 5 weeks spec	64.	15.31	{0., 43.}	77.1406	1.55871	newly 5 weeks cumul	324.	142.01	{17., 297.}	58.2747	0.544445	newly 60 weeks cumul	489.	173.61	{17., 372.}	64.6728	0.651268
28	newly 3 weeks spec	52.	83.63	{52., 116.}	66.3269	0.204223	newly 3 weeks cumul	108.	202.05	{123., 360.}	89.9907	0.247449	newly 5 weeks spec	42.	93.37	{39., 123.}	135.262	0.423564	newly 5 weeks cumul	207.	389.43	{286., 521.}	89.9275	0.26636	newly 63 weeks cumul	611.	809.39	{705., 898.}	38.5352	0.149178
29	newly 3 weeks spec	39.	54.39	{40., 68.}	40.9487	0.142389	newly 3 weeks cumul	112.	150.78	{123., 184.}	34.875	0.125262	newly 5 weeks spec	44.	53.86	{40., 67.}	27.5455	0.102664	newly 5 weeks cumul	200.	260.49	{209., 312.}	31.025	0.113833	newly 56 weeks cumul	440.	601.4	{552., 662.}	37.3682	0.137121
30	newly 3 weeks spec	46.	35.33	{11., 59.}	37.1957	0.33399	newly 3 weeks cumul	145.	121.73	{60., 177.}	27.0966	0.167799	newly 5 weeks spec	43.	23.59	{0., 47.}	50.0233	0.624766	newly 5 weeks cumul	245.	175.71	{80., 259.}	32.8857	0.225909	newly 57 weeks cumul	463.	257.05	{86., 376.}	46.581	0.351185
31	newly 3 weeks spec	17.	43.38	{28., 59.}	156.471	0.393572	newly 3 weeks cumul	48.	121.41	{88., 153.}	153.771	0.393872	newly 5 weeks spec	16.	45.18	{28., 65.}	182.5	0.432111	newly 5 weeks cumul	108.	210.5	{153., 271.}	96.3704	0.285045	newly 58 weeks cumul	420.	509.1	{392., 587.}	24.0571	0.0921344
32	newly 3 weeks spec	35.	49.97	{34., 66.}	47.6286	0.162105	newly 3 weeks cumul	65.	121.17	{88., 154.}	87.1538	0.259729	newly 5 weeks spec	21.	64.51	{46., 84.}	208.048	0.477591	newly 5 weeks cumul	124.	245.98	{190., 321.}	99.4355	0.292669	newly 60 weeks cumul	460.	697.09	{495., 797.}	53.1109	0.182121
33	newly 3 weeks spec	48.	91.26	{63., 115.}	91.0417	0.271008	newly 3 weeks cumul	140.	214.63	{153., 269.}	55.05	0.184302	newly 5 weeks spec	62.	94.23	{67., 113.}	52.4677	0.17719	newly 5 weeks cumul	272.	407.28	{317., 489.}	50.5515	0.173028	newly 63 weeks cumul	628.	774.33	{681., 864.}	24.543	0.0941293
34	newly 3 weeks spec	16.	16.62	{0., 36.}	74.	0.607995	newly 3 weeks cumul	68.	65.08	{16., 121.}	49.1176	0.301782	newly 5 weeks spec	22.	8.99	{0., 25.}	68.3182	1.10048	newly 5 weeks cumul	123.	86.99	{16., 164.}	45.4065	0.367989	newly 55 weeks cumul	356.	106.97	{16., 217.}	69.9522	0.707723
35	newly 3 weeks spec	73.	56.96	{30., 83.}	31.0411	0.205876	newly 3 weeks cumul	170.	144.8	{84., 203.}	27.4235	0.147984	newly 5 weeks spec	62.	59.45	{17., 95.}	37.9516	0.237263	newly 5 weeks cumul	317.	265.07	{139., 369.}	25.	0.143368	newly 63 weeks cumul	588.	539.62	{294., 807.}	31.1327	0.17196
36	newly 3 weeks spec	88.	28.22	{0., 55.}	68.25	0.842447	newly 3 weeks cumul	210.	140.09	{30., 237.}	41.3667	0.333964	newly 5 weeks spec	57.	12.19	{0., 28.}	78.7544	1.34354	newly 5 weeks cumul	377.	171.74	{34., 291.}	55.0557	0.47891	newly 64 weeks cumul	488.	187.44	{34., 320.}	61.709	0.552574
37	newly 3 weeks spec	41.	35.53	{21., 51.}	26.561	0.140952	newly 3 weeks cumul	138.	108.65	{70., 146.}	24.1667	0.131298	newly 5 weeks spec	36.	28.74	{16., 40.}	27.3889	0.159475	newly 5 weeks cumul	224.	169.75	{113., 226.}	25.8527	0.142276	newly 54 weeks cumul	482.	309.58	{215., 397.}	35.8008	0.205537
38	newly 3 weeks spec	82.	69.06	{49., 90.}	20.4634	0.106704	newly 3 weeks cumul	203.	172.25	{126., 205.}	18.2217	0.0924114	newly 5 weeks spec	63.	75.22	{57., 92.}	28.0317	0.11333	newly 5 weeks cumul	360.	323.15	{249., 385.}	13.9639	0.070322	newly 62 weeks cumul	525.	725.61	{509., 815.}	42.2152	0.153427
39	newly 3 weeks spec	46.	50.38	{32., 70.}	25.6087	0.102019	newly 3 weeks cumul	116.	135.67	{90., 179.}	26.5776	0.0996268	newly 5 weeks spec	36.	53.12	{35., 67.}	51.2222	0.175027	newly 5 weeks cumul	202.	242.18	{168., 314.}	26.703	0.10073	newly 58 weeks cumul	447.	632.65	{478., 705.}	44.9866	0.16177
40	newly 3 weeks spec	52.	41.62	{0., 88.}	54.4615	0.571592	newly 3 weeks cumul	151.	157.66	{25., 290.}	51.2715	0.302684	newly 5 weeks spec	41.	19.72	{0., 46.}	69.6585	0.9566	newly 5 weeks cumul	250.	207.34	{30., 379.}	44.872	0.331362	newly 64 weeks cumul	483.	247.51	{30., 401.}	54.793	0.482952
41	newly 3 weeks spec	64.	83.83	{59., 106.}	35.6719	0.128873	newly 3 weeks cumul	223.	204.09	{142., 257.}	15.6457	0.0775595	newly 5 weeks spec	76.	85.72	{68., 105.}	17.5263	0.0691864	newly 5 weeks cumul	393.	381.19	{298., 448.}	12.2163	0.0572617	newly 61 weeks cumul	574.	756.3	{629., 835.}	32.0105	0.118494
42	newly 3 weeks spec	30.	43.69	{23., 75.}	60.8333	0.257269	newly 3 weeks cumul	92.	127.49	{75., 195.}	51.2717	0.178438	newly 5 weeks spec	39.	38.84	{7., 61.}	36.6667	0.317574	newly 5 weeks cumul	182.	208.56	{133., 331.}	33.3956	0.148615	newly 61 weeks cumul	534.	456.94	{242., 638.}	26.412	0.178339
43	newly 3 weeks spec	35.	42.68	{28., 57.}	31.7714	0.119958	newly 3 weeks cumul	108.	117.72	{85., 146.}	18.6852	0.0771386	newly 5 weeks spec	36.	41.54	{24., 55.}	27.7778	0.112008	newly 5 weeks cumul	191.	202.43	{145., 249.}	16.1518	0.0686488	newly 54 weeks cumul	401.	476.75	{412., 546.}	21.8828	0.0870561
44	newly 3 weeks spec	64.	43.13	{24., 63.}	36.0781	0.217809	newly 3 weeks cumul	144.	104.83	{66., 143.}	32.3264	0.182195	newly 5 weeks spec	60.	53.27	{27., 81.}	28.7167	0.162689	newly 5 weeks cumul	278.	208.84	{123., 292.}	29.223	0.167673	newly 62 weeks cumul	520.	610.17	{396., 807.}	32.4481	0.140396
45	newly 3 weeks spec	36.	53.88	{31., 73.}	61.7778	0.244196	newly 3 weeks cumul	140.	149.53	{95., 197.}	23.55	0.113394	newly 5 weeks spec	47.	47.38	{18., 72.}	32.	0.223809	newly 5 weeks cumul	246.	248.64	{152., 330.}	22.5528	0.120604	newly 59 weeks cumul	529.	484.89	{279., 669.}	27.9112	0.165184
46	newly 3 weeks spec	25.	32.75	{18., 49.}	44.52	0.159008	newly 3 weeks cumul	61.	83.9	{53., 118.}	43.3443	0.146614	newly 5 weeks spec	28.	36.01	{17., 56.}	45.1071	0.17132	newly 5 weeks cumul	125.	154.51	{95., 215.}	34.984	0.129661	newly 58 weeks cumul	517.	501.35	{370., 642.}	17.8356	0.0854556
47	newly 3 weeks spec	97.	71.13	{34., 103.}	31.0206	0.22131	newly 3 weeks cumul	190.	157.2	{81., 229.}	27.6105	0.174872	newly 5 weeks spec	95.	91.44	{63., 118.}	19.7684	0.154781	newly 5 weeks cumul	400.	334.11	{207., 448.}	22.4675	0.148115	newly 64 weeks cumul	577.	731.25	{496., 880.}	37.0763	0.176501
48	newly 3 weeks spec	31.	53.81	{34., 76.}	78.9355	0.264837	newly 3 weeks cumul	100.	156.58	{112., 203.}	59.9	0.205101	newly 5 weeks spec	36.	43.83	{23., 64.}	40.5278	0.187557	newly 5 weeks cumul	184.	250.02	{178., 335.}	42.2391	0.16052	newly 58 weeks cumul	364.	430.93	{284., 587.}	30.7005	0.134309
49	newly 3 weeks spec	70.	56.98	{28., 80.}	25.4	0.147938	newly 3 weeks cumul	184.	142.95	{90., 195.}	25.3098	0.142698	newly 5 weeks spec	55.	66.79	{39., 92.}	37.8364	0.158915	newly 5 weeks cumul	319.	273.26	{159., 345.}	19.9561	0.110802	newly 62 weeks cumul	565.	686.95	{428., 806.}	32.3664	0.138642
50	newly 3 weeks spec	37.	33.87	{13., 54.}	33.0541	0.185267	newly 3 weeks cumul	82.	82.56	{37., 122.}	31.8537	0.155644	newly 5 weeks spec	52.	41.67	{17., 65.}	31.3654	0.21094	newly 5 weeks cumul	186.	162.63	{75., 239.}	28.1882	0.159126	newly 60 weeks cumul	449.	594.16	{378., 754.}	42.6459	0.174109
